# A model for the promotion of consumption of locally available indigenous vegetables among primary school children

**DOI:** 10.3389/fnut.2024.1394905

**Published:** 2024-08-13

**Authors:** Hlekani Vanessa Mbhatsani, Betrand Ayuk Tambe, Mthokozisi Kwazi Zuma, Xikombiso G. Mbhenyane

**Affiliations:** ^1^Division of Human Nutrition, Faculty of Medicine and Health Sciences, Stellenbosch University, Cape Town, South Africa; ^2^Department of Nutrition, Faculty of Health Sciences, University of Venda, Thohoyandou, South Africa

**Keywords:** model, promotion, vegetables, children, indigenous

## Abstract

**Introduction:**

The study’s primary aim was to develop a model for promoting the consumption of selected locally available indigenous vegetables for primary school children. Model development was phase three of a bigger study.

**Methods:**

A qualitative approach was used for this phase. The model was developed following three steps: model design, development, and validation. The iterative phases of model development starting with data preparation, data understanding, model assembly, model audit and model delivery were adopted.

**Results:**

The model is influenced by the World Health Organization’s approaches (medical, educational, behavioural change, empowerment, and societal change approaches). The researchers synthesised the data from phases one and two of the study and selected content which suited the model. Two experts’ engagement consultations were conducted for inputs: one face-to-face and one virtual. This was followed by model validation from the end users.

**Discussion:**

The model developed for this study proposes that, for the promotion of locally available indigenous vegetables within the primary school setting, the following critical issues should happen: i) a medical approach should be in place; ii) education or correct information should be provided; iii) behavioural change should be enabled; iv) empowerment should be provided, and v) societal enhancement should be encouraged. Furthermore, it suggests that the promotion of the consumption of locally available vegetables among primary school children can be achieved when the identified stakeholders work together.

## Introduction

According to Abrahams et al. (1), South Africa has seen wide-reaching changes in diet, nutritional status, disease patterns, and life expectancy among children and adults. These changes are thought to occur as three separate transition processes: the demographic, epidemiological and nutrition transition. Biro and Wien (2) reported that these transitions - associated with rapid socio-economic changes - have increased the risk of obesity in both childhood and adulthood. This transition is influenced by the Western-type diet that is characterized by excessive consumption of total and saturated fat, a high intake of sugar-sweetened beverages, as well as a low intake of fiber, legumes, and fruit and vegetables which predisposes many to chronic degenerative diseases such as Type II diabetes, cardiovascular disease, and some cancers in later life (3).

Eating habits are a part of one’s lifestyle and the dietary pattern of adolescents is one of the major public health concerns because there is a direct link between poor diet and chronic diseases (4). Poor eating habits are formed and established from childhood and adolescence so if there is no intervention to correct it, the dietary habits can be the source of health problems in the future. Without adequate diversity and meal frequency, infants and young children are vulnerable to undernutrition, especially stunting and micronutrient deficiencies, and to increased morbidity and mortality. Promoting healthy dietary habits in childhood and adolescence is necessary to prevent diet-related diseases. Intervening at the basic school level is crucial because adopting unhealthy dietary habits tends to worsen with adolescence (5).

Vegetables and fruit are often neglected, contributing to a monotonous diet in many South African households (6, 7). It is well known that consuming sufficient amounts of fruit and vegetables reduces the risk of disease (8, 9). It has been argued that African leafy vegetables can play an essential role in the World Health Organization’s (WHO) global initiative on consuming fruit and vegetables (10). South Africa has plenty of local indigenous foods including fruits and vegetables in all provinces that consumers can select from. However, data confirms that these indigenous options are neglected and regarded as food for the poor and inferior. The decline in the use of indigenous vegetables and fruits contributes to nutritional deficiencies, especially among children in rural areas. Adequate intake of vitamins and minerals is essential for controlling and preventing common micronutrient deficiencies such as vitamin A, iron, and iodine. Most rural people do not earn a regular income and cannot purchase exotic vegetables even if these are available (11). A recommendation from the South African National Food Consumption Survey (1999) for children 1–9 years where it was suggested that the sustainable use of indigenous vegetables and fruits should be encouraged in the appropriate setting (12).

According to Vorster et al. (13), the role of leafy vegetables in South African households is highly variable and depends on aspects such as poverty status, the extent of urbanization, distance from fresh produce, and time of the year. There are differences in the consumption, perception and acceptability of indigenous foods and African leafy vegetables among different age groups and across districts in South Africa. Factors that contribute to decisions about indigenous food consumption cannot be ignored. These include, among others, sensory characteristics of food, such as appearance, smell, texture, taste, and social class (14). In some instances, a lack of variety in cooking methods (15) of traditional vegetables could make them less attractive and reduce the likelihood of their consumption. The inclusion and consumption of these indigenous and traditional food plants especially African leafy vegetables have also been affected negatively by colonization and the attempted assimilation of people from the traditional cultural practices.

So many food-based strategies such as National School Nutrition Program (NSNP), food-based dietary guidelines, Value-Added Tax exclusion and the Roadmap to Health booklet are available to promote the consumption of fruit and vegetables across all population groups in South Africa. However, consumption of these indigenous foods remains very low among primary school children (16). Assessment of school-age children’s nutritional status and dietary patterns is crucial to identify, design, and implement relevant interventions. These interventions are necessary because promoting healthy dietary habits in childhood and adolescence is linked to preventing diet-related diseases.

Model development is an iterative process in which many models are derived, tested, and built upon until a model fitting the desired criteria is built (17). There are five steps of model development Baer (18): model design, data engineering, model assembly, model validation, and model implementation articulated. According to Nilsen (19), theories, models, frameworks, and approaches are distinct concepts; however, researchers use these terms interchangeably sometimes, especially in implementation science or studies. Model development also aids them in describing, predicting, testing, or understanding complex systems or actions. Accordingly, models often provide a context for conducting research and might consist of actual items or abstract forms, such as sketches, mathematical formulas, or diagrams (20).

Two major types of models are qualitative and quantitative, which are further deduced into deductive and inductive models. In our study, the model followed the inductive type which normally takes a “bottom-up” approach that starts with specific observations and measures, then continues with the identification of patterns and regularities, therefore formulating some tentative hypotheses that can be explored and resulting in general conclusions or theories (21, 22). Developing this model was done in line with the primary aim of the study which was to develop a model for the promotion of the consumption of selected locally available indigenous vegetables by primary school children. This model attempts to answer the research question of whether regular inclusion of locally available indigenous vegetables in the NSNP menu can promote their overall consumption.

## Materials and methods

This study aimed to develop a model for promoting the consumption of locally available indigenous vegetables among primary school children. Model development was phase three of a quasi-experimental study design with a non-equivalent (pre-test/post-test) control group design wherein the school children were selected without random assignment into a control or treatment group. The two schools were purposefully selected based on previous research findings in the same schools (16). These were the study phases: (i) development of the intervention, (ii) implementation of the intervention, and (iii) development of the model. The research design for this phase was qualitative. Data was collected from 287 primary school children, whereby 151 were from intervention school and 131 were from the comparison school. The intervention was only implemented at the intervention school.

The model development phases as introduced by Grolemud and Wickmann (23), Hall et al. (24), and Biecek (25) including business understanding, data understanding, data preparation, modeling, evaluation, and deployment were employed. This research adopted the iterative phases of model development starting with data preparation, data understanding, model assembly, model audit and model delivery and excluded business understanding (24, 25) because the business understanding and preparation had already been concluded.

Model development (Phase-three) was divided into the synthesis of the data from phases one and two to select suitable constructs, end-users face-to-face expert engagement (academics, chefs, dieticians, nutritionists, department of Health representatives, agriculture representatives, civil society organizations, and postgraduate students) and virtual consultation workshop for model validation (for inputs) and validation by end-users. In the following paragraphs, the steps followed in the development of the model are described:

### Step 1: model design

Model design defines the model’s overall structure, such as what shall go in and what shall come out of it (18), what the model should do, and what precision, accuracy or performance is needed (26). This step is also known as problem description Schaus et al. (26), where all the requirements are analyzed first. In our study, model design encompasses data understanding and data preparation. The data understanding and trial steps were conducted simultaneously. The research team consisting of the study promoter, researcher, statistician, and postdoctoral fellow held a workshop to make sense of the research findings. In this workshop, the group explored and interpreted the data to select suitable variables to be fitted into the model.

The WHO’s Health Promotion Model (HPM) was used to support the design and development of this model. The HPM model is integrated into the chosen model because of the way it classifies the determinants of health into three components: (a) individual characteristics and experiences, (b) behavior-specific cognitions and effects, and (c) situational/interpersonal influences (27). These components became the overarching guiding principles through which the variables that could fit into the five WHO approaches were allocated.

Throughout the model’s design in this study, the WHO approaches are used in conjunction with the HPM. The model design will highlight how the HPM knowledge, attitude, and practice (KAP) approach is applicable as it is used to promote and evaluate an increase in the knowledge, attitude and practices or behaviors of targeted individuals, groups, and communities. The KAP approach has three key steps: (1) provision of new knowledge, (2) acceptance of and the development of a positive attitude toward the new knowledge, and 3 an intention to take action to change a related behavior or practice (28).

The model development process and specific data that was fitted in the model are reported below following the five approaches of the WHO and HPM model.

#### Medical approach

Baseline and post-intervention information was collected through a survey from 252 primary school children. This exercise is aligned with the WHO’s Medical Approach (MA). It allowed the researchers to order all the necessary data that helped them describe the study population. HPM notes that individual characteristics are crucial to understanding the subjects. Individual characteristics and experiences are intrinsic factors such as (gender, age, genetics) and experience factors that inform future behavior. The MA in this study guided the screening of socio-economic parameters of children, taking anthropometric measurements of weight and height, body composition, and the determination of micronutrient status through biochemical measurements.

#### Educational approach

This study adopted the Educational Approach (EDA) to apply the first two steps of the KAP approach, namely (1) provision of new knowledge and (2) acceptance of and the development of a positive attitude toward the new knowledge as reported by Corcoran (28) whereby formal and informal education and training was done. The researcher used lessons and group discussions to sensitize and advocate for children to create a positive attitude toward locally available foods and their role in nutritional health. The researcher also used the lessons to prepare the children for the possibility of these foods being included in their school meals. Group discussions and demonstrations where children shared experiences about the consumption of indigenous foods were used as a platform for learning. The study also provided lessons and demonstrated the preparation of vegetable dishes as a teaching aid. Furthermore, the study conducted a sensory evaluation where the researchers could assess the acceptance of and the development of a positive attitude toward the new knowledge by determining how children reacted after tasting the intervention dishes. This decision is supported by Laverack (29), who depicted that knowledge could lead to a greater sense of disempowerment when a person cannot use the new information they have acquired to improve their circumstances. In this study, lessons were integrated with the provision of dishes prepared from locally available indigenous vegetables (*Amaranthus*, *Bidens pilosa*, and *Vigna ungucuilata*), which bodes well with the third part of the KAP approach of an intention to take action to change a related behavior or practice.

The researcher provided training to the Volunteer Food Handlers (VFHs) on preparing and rationing the NSNP meals. The VFHs accepted and developed a positive attitude toward the training by consistently following the recipes provided to them. They embraced the new knowledge by adhering to the NSNP meal schedule without asking the research team. They also sacrificed extra time to prepare dishes that required more time than the usual NSNP meals that they usually cooked.

School children were also trained to serve the meals to their peers; based on the observations by class teachers and the research assistant team, they served the food per serving size – revealing that nutrition education was successfully implemented and executed. The principal and school governing body provided an enabling environment for education by integrating the lessons into an existing Life Skills Orientation module without disturbing the entire school timetable.

#### Behavioral change approach

This study used the three steps of the KAP, namely: (1) provision of new knowledge; (2) acceptance of and the development of a positive attitude toward the new knowledge, and (3) an intention to take action to change a related behavior or practice (28) and related this to the behavioral change approach (BCA). New knowledge involved the infusion of locally available indigenous vegetables as part of the NSNP meal. Previous research established that primary school children do not like locally available indigenous vegetables.

#### Empowerment approach

Empowerment is an integral part of the WHO’s definition of health promotion as it delineates people’s aptitude to increase control over their health. Empowerment, therefore, encompasses access to information and the skills to use such data in practice. And having the opportunity and power to use the information to make desired changes in one’s life. Empowerment is closely linked to engagement and participation (30). Empowerment of individuals is better identified by its three elements: information, attitudes, and skills. To advance empowerment and make decisions informed by knowledge, people need to have the correct knowledge, the attitude that endorses self-belief and efficacy, and the skills to put their knowledge into practice in diverse settings (30).

#### Societal change approach

According to Borelli et al. (31), creating awareness campaigns that promote diet diversification and the nutritional, environmental and economic benefits of orphan crops and wild edible species could encourage societal change. Three locally available vegetables were promoted, and the economic benefit was activated through supporting local small-scale farmers who provided the vegetables throughout the implementation. Furthermore, Naidoo and Wills (30) highlight that making healthy choices easier denotes how the environment, social networks, sense of security, socio-economic circumstances, families, and resources individuals have in their local neighborhood can affect their health.

### Step 2: model development

Model development in this study means model assembly (24, 25). This is the heart of model development where the internal architecture of the model is defined. First, the raw data was transformed into an equation for mathematical models, a figure, a framework, or any type of technique used based on the purpose and the type of the model being built (18, 26). Once the work from the research team was completed, the second step was experts’ consultation workshop held in Cape Town where chefs, dieticians, researchers, food policy personnel, health government representatives from both national and provincial levels, and members of civil society attended. During this workshop, the researcher shared her findings with the experts. The workshop’s outcomes included further identifying results that could suit the model and selecting and prioritizing the elements of the model. The development of the model for promoting the consumption of indigenous vegetables among primary school children considered that all stakeholders and participants form part of the decision-making process. The final step was for the researcher and team to fine-tune the parameters before final assembly and variables fitted into the model. A graphic designer form Stellenbosch University was the consulted who created visual text and imagery.

### Step 3: model implementation

Model implementation in this study refers to the proposed model implementation plan as determined by the information fitted into the model, identified actors and stakeholders and their roles or responsibilities.

### Step 4: model validation

Model validation in this study refers to model audit and delivery (24, 25). According to Schaus et al. (26), validation is a testing step for the model. It is an independent review and assertion of the model’s fitness for use. One of the ways in which models are validated is through expert appraisal, a technique to get suggestions for material improvement. By conducting an assessment by experts and getting suggestions for improvement of the model developed, then revised according to expert advice. Expert assessment is expected to make the model tools and outcomes more precise and effective and has high techniques (32).

The researcher adopted a validation tool with questions like model understandability, translation, acceptability, implementation, practicability, integration, and political buy-in. The research team then organized the second expert consultation workshop held virtually via Microsoft Teams. The proposed model was presented to some of the experts who attended the face-to-face workshop and some new experts who work in the food and agriculture in Uganda (Nutritionist), Ethiopia (Dietitian working for UNICEF), and Nigeria (academia) as well as researchers, representatives from the department of health, chefs, and dieticians from South Africa. The primary aim of this workshop was to obtain buy-in and input on the final model. The secondary purpose was to test the applicability and acceptability of the model within experts’ institutions and their opinions regarding the validation instrument itself. The researcher then consolidated all inputs and sent the information to the Graphic Design Department at Stellenbosch University (SU), who designed the model as infographics.

### Ethical considerations

The protocol was submitted to the Stellenbosch University Health Research Ethics Committee (HREC) under registration number #S17/09/165. Approval was sought from the Department of Basic Education of the Limpopo Province and the primary school head or School Governing Body before commencement. Ethical principles relating to human subjects were adhered to during the study as described in the subsequent sections. Experts signed a consent form and confidentiality agreement.

### Data analysis

Data from the research team workshop, face-to-face expert consultation, virtual expert workshop and end-user validation was analyzed following the six steps described by Creswell (33). These are the steps in their chronological order: organizing and preparing data, reading or looking at all the data, data coding, generating a description and themes, representing the description and themes and interpreting the findings. ATLASti.8 software generated a description and themes for data coding. Thematic analysis was used whereby similar information was clustered into themes and sub-themes. The information was interpreted and compared with relevant literature.

## Results

The findings are reported according to the model development process, as highlighted in the methodology of this paper.

## Model design and development

### Fitting data into the model

**Figure 1:**
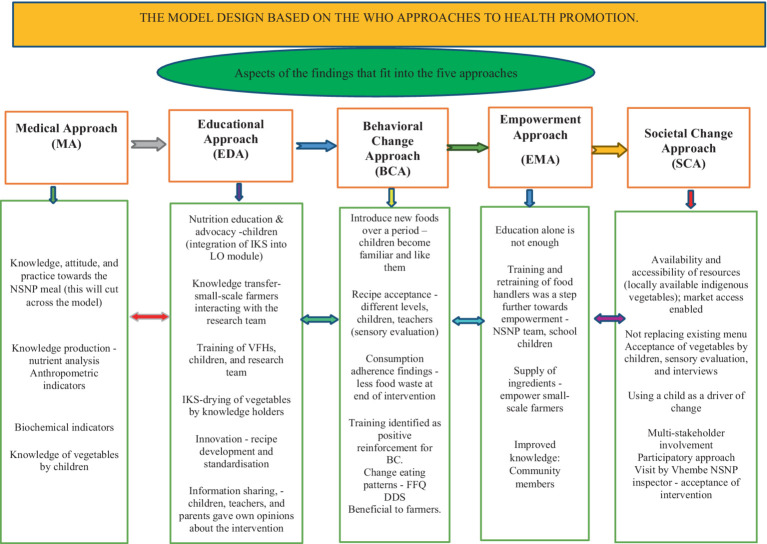
The model design based on the World Health Organization’s approaches to health promotion.

The data synthesized from phase 1 and phase 2 of the study were fitted into these five approaches, as summarized in Figure 1. The model developed for this study proposes that, for the promotion of locally available indigenous vegetables within the primary school setting, the following critical issues should happen: (i) a medical approach should be in place; (ii) education or correct information should be provided; (iii) behavioral change should be enabled; (iv) empowerment should be provided, and (v) societal enhancement should be encouraged.

The data from the medical approach showed that some constructs improved 5 months after intervention with the three dishes. The data also revealed that nutrition lessons and training provided to the children and volunteer food handlers could have contributed to increased consumption of these vegetables by children in the intervention school.

**Figure 2:**
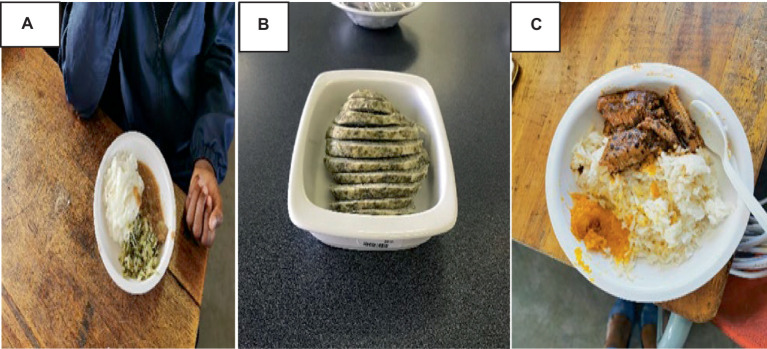
Innovative dishes for the intervention.

The chef used innovative ways to prepare meals for the children using the food items already included in the school menu (see Figure 2) *Amaranthus* vegetable was added to cabbage (A: *Amaranthus* cabbage dish), dumpling (steam bread) was prepared using *Bidens pilosa* vegetable as a condiment (B: *Bidens* dumplings dish), and pilchards in tomato were mixed with cowpea leaves (C: Fishpea dish) (34).

These dishes were liked by children at the intervention school and were suitable for infusion into the school meals. The findings of sensory evaluation in the intervention school showed that children accepted the meals. The results further designate good adherence and less plate waste of the intervention dishes by children over the 5 months period.

The empowerment approach in this study is supported by the findings that revealed that VFHs possessed the necessary skills and abilities to prepare the NSNP dishes based on the training offered and could interpret the menu and follow the feeding schedule thus displaying empowerment. Children formed part of the intervention program by serving their peers the required meals as shown to them. The researcher did not educate parents and guardians about the preparation of the dishes; however, children and VFHs shared the information with them. Some children managed to keep a bit of dumpling to give their parents to taste. Some of the parents reported that they needed lessons on how to prepare meals the way the intervention meals were cooked.

This study promoted dietary diversity by providing meals prepared from *Amaranthus*, *Bidens Pilosa,* and cowpea - three locally available vegetables. The economic benefit was activated through supporting local small-scale farmers who provided the vegetables throughout the implementation of the intervention. In addition, peers, parents, schoolteachers, and representatives from the Vhembe district NSNP team were receptive to the initiation and accepted the intervention meal. All these could easily promote and enhance societal change.

## Model benefits, barriers, and end users

**Figure 3:**
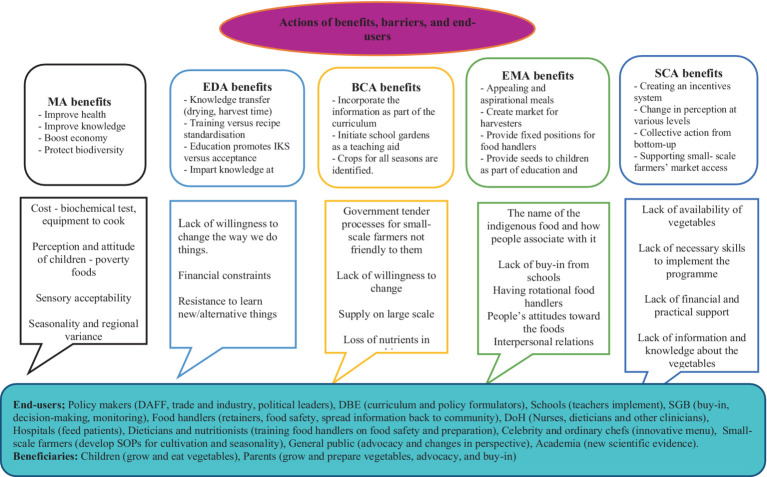
Beneficiaries and actors in the proposed model.

Once the aspects that fit into the model (Figure 1) were identified and allocated, the action benefits, barriers, and possible end users were identified and put together in (Figure 3) below. Some of the benefits included that the Medical Approach can improve general and nutritional health, the Educational Approach can facilitate knowledge and skills transfer, the Behavioral Change Approach can inform initiation of school gardens as part of the curriculum, the Empowerment Approach can benefit through the creation of opportunities for small-scale farmers to supply ingredients that chefs can use to develop appealing and aspirational meals. Societal Change Approach benefits can include changes in perception at various levels. Children and parents were identified as beneficiaries. Meanwhile, end-users were clustered into policymakers, implementers (schools, celebrities, and ordinary chefs), the public, and academia.

## Model implementation plan

**Figure 4:**
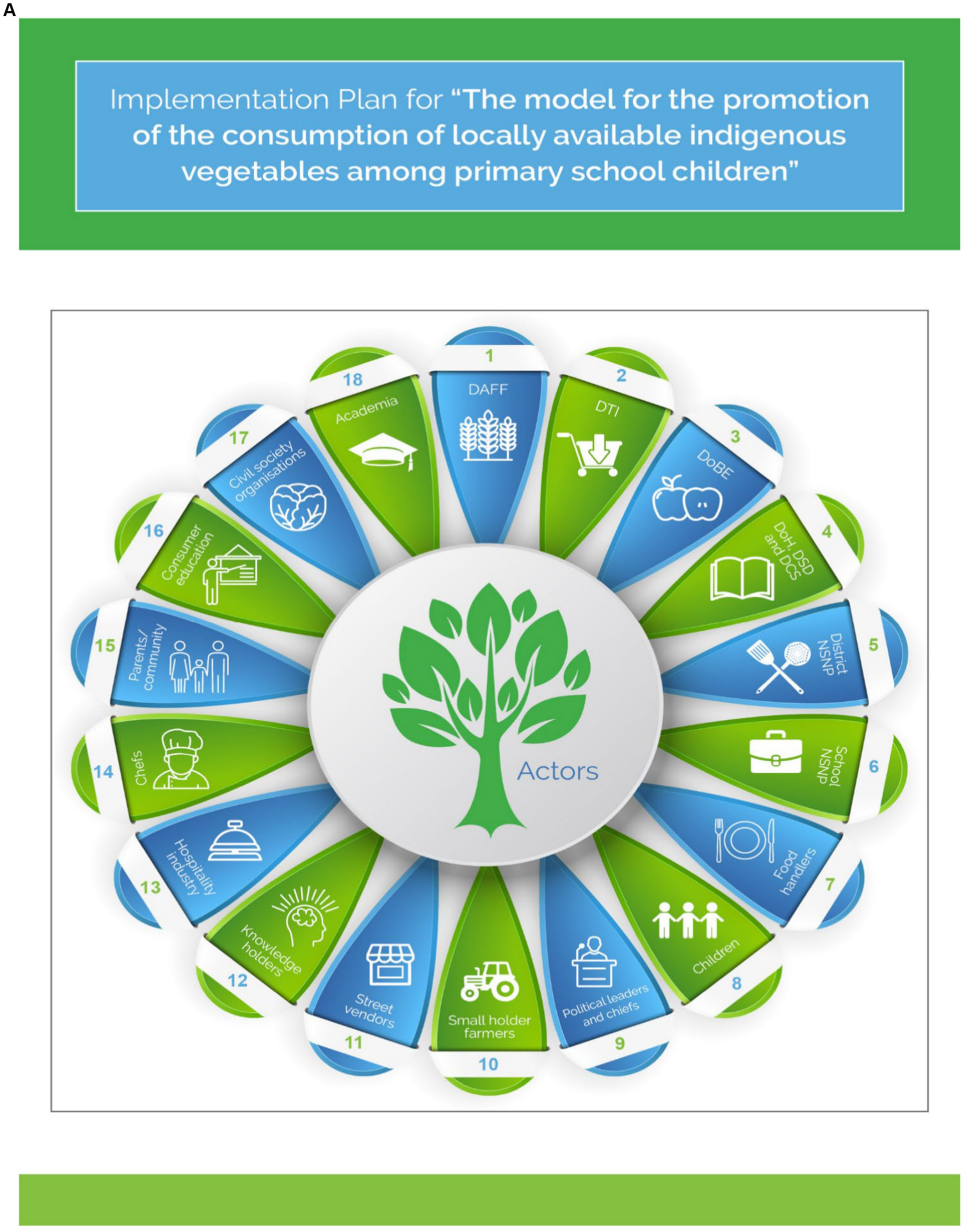

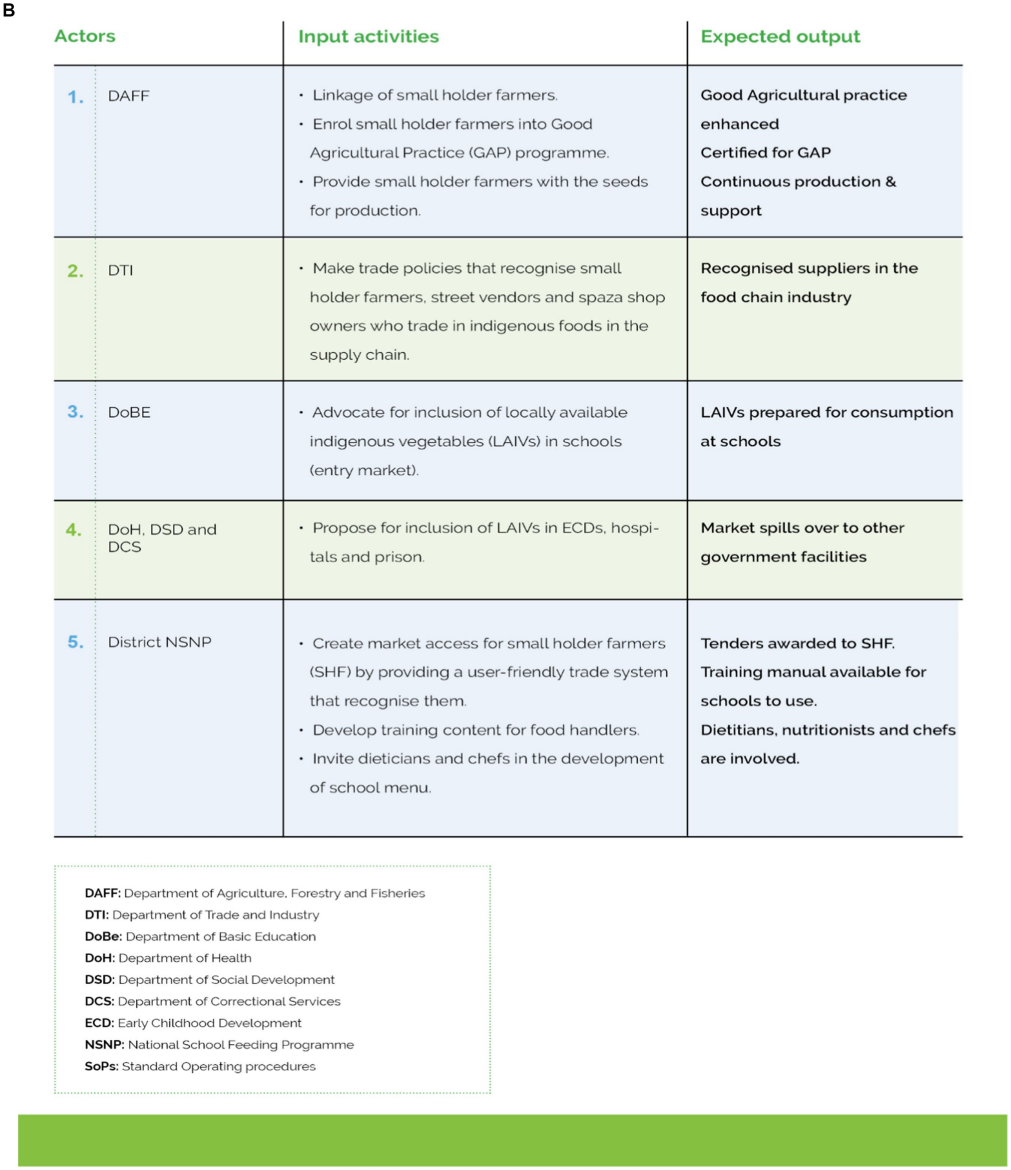

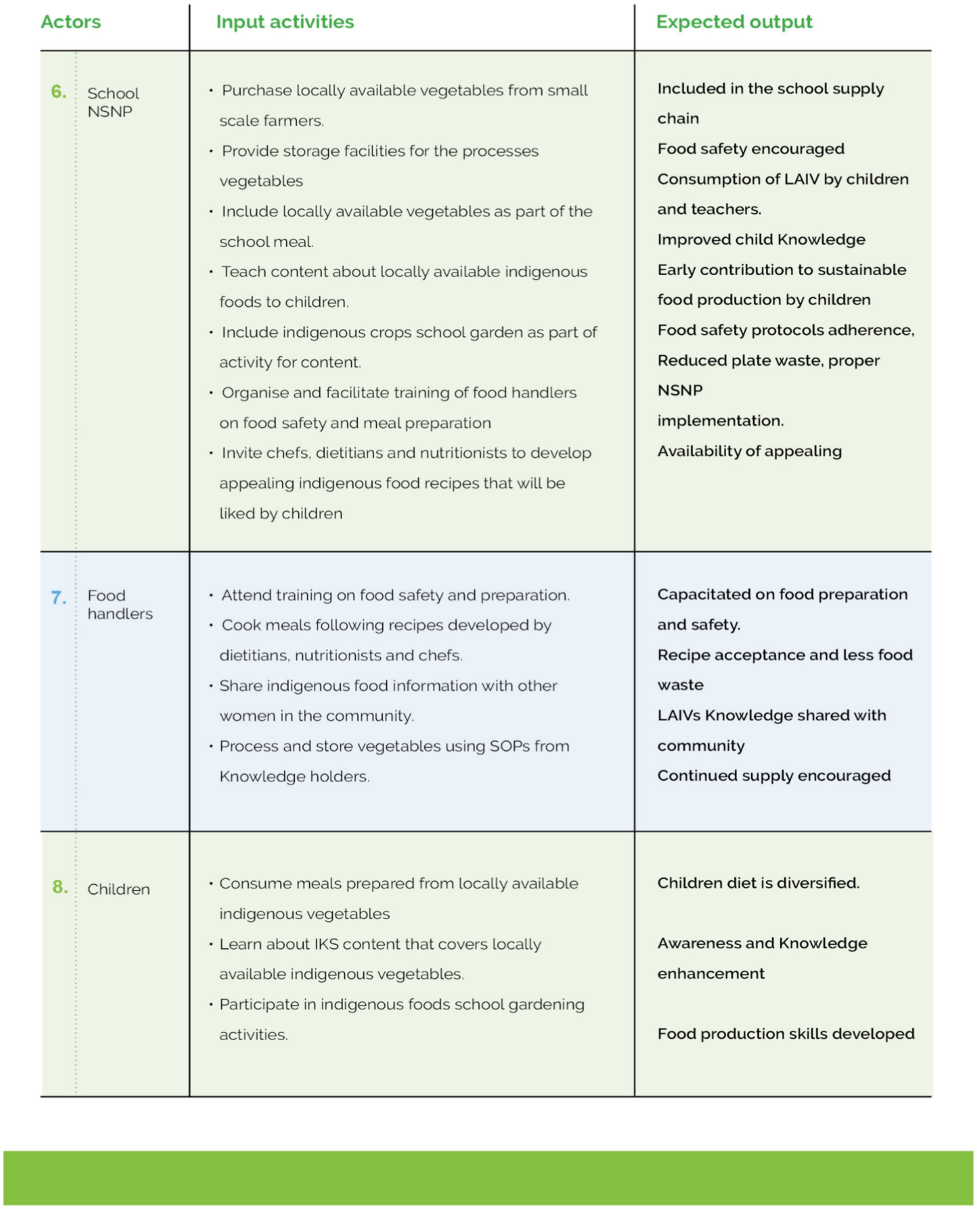

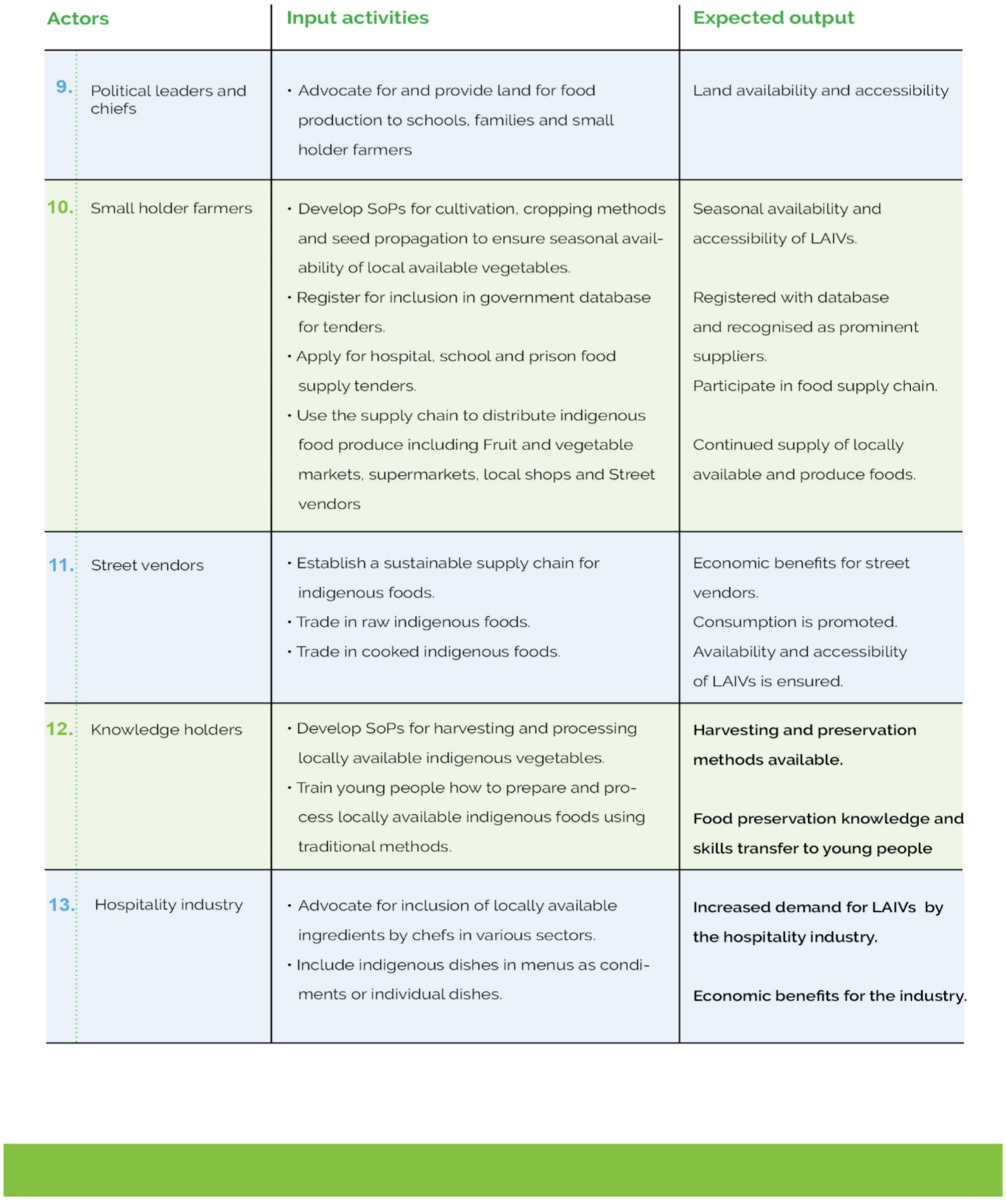

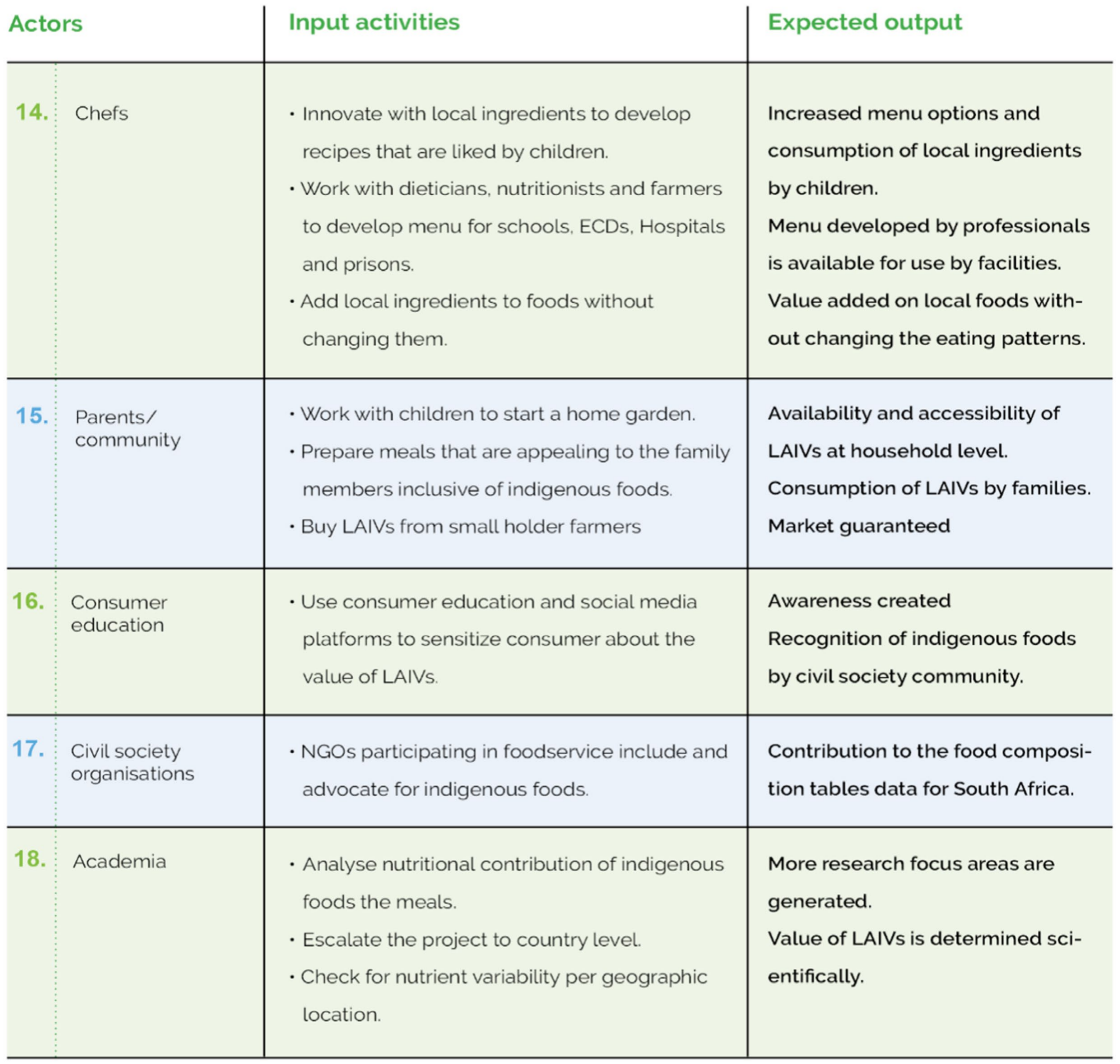
The model for the promotion of Indigenous foods. Part **A**: Actors in the implementation of the model. Part **B**: Action to be taken by each actor in the implementation of the model.

The final model for promoting the consumption of indigenous foods among young children is presented in infographics in Figure 4. This model is divided into two parts; the first page presents a cycle showing all the actors – each with a number (part A). The second, third, fourth and five pages have the actor’s numbers, the actions they should take, and the expected outputs (Part B).

The model suggests that the promotion of the consumption of locally available vegetables among primary school children can be achieved when the identified stakeholders work together. The developed model proposes a list of possible implementers or stakeholders, input activities, and expected outputs. The stakeholders are divided into five categories: (i) policymakers; (ii) industry; (iii) community; (iv) academia, and (v) civil society organizations.

Food production can be sustainable if policymakers such as the Department of Agriculture, Forestry and Fisheries (DAFF) assist small-scale farmers through capacity development activities including the Good Agriculture Practices (GAP) program and seeds provision. In addition, if the Department of Trade and Industry (DTI) makes trade policies that recognize small-scale farmers and local *spaza*^1^ shops as part of the food supply chain small-scale farmers will become recognized suppliers and the DBE can be used as the entry market.

The model further articulates that once the DBE has given small-scale farmers market access, other government departments such as the Department of Health (DoH), Department of Social Development (DSD), and the Department of Correctional Services (DCS) can provide the same access through their facilities (hospitals, early childhood development (ECD) centers, and prisons). Enabling market access for small-scale farmers can have economic benefits and, in turn, increase access and availability of locally available vegetables to the community thereby promoting their consumption. Political leaders and chiefs can make land accessible and available to small-scale farmers, the community, and schools. Small-scale farmers can (after being capacitated by DAFF), develop Standard Operating Policies (SOPs) for cultivating and processing the local vegetables, registering a company, developing a business plan, registering for inclusion in the food supply chain, and applying for school, prison, or hospital food supply tenders, and using the supply chain to distribute locally available indigenous vegetables to local shops, street vendors, and families.

The Vhembe District NSNP in this study was represented by school NSNPs, VFHs, and children with their contribution to the development of this model. Contribution to the implementation of this model by these representatives mentioned above suggests that the district NSNP would develop training content for VFHs and create market access for small-scale farmers by providing a user-friendly trade system that recognizes them. Meanwhile, the VFHs will cook meals following the recipes developed by dieticians, nutritionists, and chefs who use locally available indigenous vegetables.

The model also suggests that the hospitality industry can promote the consumption of locally available vegetables by, for example, chefs advocating for the inclusion of local ingredients in the various hospitality industries. Moreover, street vendors can promote locally available vegetables by selling them. Chefs can also work with dietitians, nutritionists, and food scientists to develop menus for schools, ECD centers, and prisons by innovating with ingredients to develop recipes that are liked by children and that use local ingredients in local foods without changing them. The involvement of chefs and street vendors could benefit the hospitality industry economically at different levels and increase the consumption of locally available foods at the community and population levels.

The other actors involved in this model include the community/parents (buying locally available indigenous vegetables from small-scale farmers and preparing meals for everyone in the household), knowledge holders (developing SOPs for processing and harvesting locally available indigenous vegetables, training young people on how to prepare and process locally available indigenous vegetables using traditional methods), civil society organizations and academia (the nutrient analysis) who can contribute to food composition table for South Africa data that will be useful for nutrition professionals and escalate the information about indigenous foods at country level.

## Model validation

Experts indicated that the model was applicable and easy to follow; they also provided further inputs that needed to be addressed before validation by end-users. Stakeholders’ engagement at various points of the study was the most crucial step because the researcher used ideas obtained from the group discussions to make further sense of the acceptability of this model.

The researcher hand-delivered the printed model and validation forms to schools (intervention and control); for the rest of the stakeholders such as DAFF, National and provincial Department of Health, DBE and Vhembe district NSNP office an email was used to send other stakeholders the validation documents. Finally, the developed and validated model was reported to the relevant authorities once the study was completed in May and June 2023 after the report was submitted to Stellenbosch University. In this manner, the societal change impact of this research could be realized as a potential outcome could be the adoption of including these foods as part of the NSNP.

## Discussion

This model proposes that the promotion of the consumption of indigenous vegetables among primary school children should happen through schools as an entry point and then be further expanded to other government departments. The model suggests that it can be possible if DAFF can ensure that small-scale farmers are identified and supported for GAP programs and become certified. The model also proposes that once these farmers are certified, trade and industry must create an enabling environment for them to become suppliers. The model identifies the DBE as the needed trailblazer that other government departments such as DoH and DSD could emulate. This is similar to the study in Guatemala where schools function as institutional markets for the procurement of orphan crops (31).

This initiative has been effective in countries like Brazil where a joint effort between the Ministries of Environment and Social Development increases farmer incentives to continue growing and managing orphan crops – crops that can be sold via two important public procurement programs: the National School Feeding Program which establishes that at least 30% of the food purchased with federal funds must be procured directly from family farmers, and the Food Acquisition Program which pays an additional 30% for organic and agroecological produce grown by family farmers (35). Currently, small-scale farmers in South Africa are not recognized suppliers in the school or hospitality food supply chain in South Africa. This model advocates that capacity building is crucial for small-scale farmers since it could benefit them economically. It was reported in Kenya that even when market opportunities exist, small-scale farmers lack the capacity to pursue them, as very few can write a business plan or a loan application to a local microfinance institution or a commercial bank. This means that small-scale farmers lack access to a steady market for orphan crops and are excluded from value-addition opportunities that could generate extra income (31).

According to Borelli et al. (31), out of the seven countries (Brazil, Kenya, Guatemala, India, Mali, Sri Lanka and Turkey) reviewed for promoting orphan crops, the findings suggest that engagement with the gastronomy sector and celebrity chefs have also sparked new interest in these forgotten crops, mainly by urban consumers. This resulted in developing an acceptable intervention suitable for infusion into the school meals. The findings of sensory evaluation in the intervention school showed that children accepted the meals. According to Triador et al. (36) the results of the data collected from 76 of 116 children (65.5%) in Canada support that school interventions have the potential to increase vegetable and fruit consumption. The results further designate good adherence and less plate waste of the intervention dishes by children as well as positive sensory evaluation (37). The findings augur well with one of the HPM theoretical propositions which states that the greater the commitment to a specific plan of action, the more likely health-promoting behaviors will be maintained over a period (36, 38). The increased adherence to the consumption of the intervention meals by children in this research may be linked to one of the points mentioned above. In this study, teachers ate the school meal with the children in the classroom, which could have added value. The school management and school governing body members also timeously requested the meal, which was more likely to encourage children to accept and consume the meals more. According to Kedia et al. (39), humans often learn by watching others and then imitating, or modeling, what they do or say.

This model developed from this research used a bottom-up approach which was highlighted by Padulosi et al. (40); they used a decentralized approach to guide national policies toward increasing the amount of attention, funding, and technical and service support for orphan crops to make food systems more nutritious while supporting sustainable agricultural practices and links with small-scale farmers. In this study in the Vhembe District of Limpopo province, the collaboration among parents, teachers, and small-scale farmers contributed greatly to adherence to consumption by primary school children. A bottom-up approach could harness and create an enabling environment for the community to accept the model since it is a model that proposes the initiative be more collaborative and not imposed on the consumers and small-scale farmers. Similarly, in Guatemala, collaboration with the Ministry of Education, the Ministry of Public Health and Social Assistance, the Ministry of Agriculture, and local non-governmental organizations (NGOs) is leading to the greater use of orphan crops in the country’s school meal programs. Children aged 6–12 years of age receive school meals that include orphan crops to meet their daily nutrient intake (40).

In a study conducted by Nepfumbada et al. (41) expert consultation involving dieticians, social workers, and ECD managers reviewed the list of Indigenous Foods (IF) voted for to develop a diet that is suitable and acceptable for children under five in ECD centers. Their review formed a follow-up on providing knowledge of the research, what it entails, and the IF diet that will be developed for implementation. After being reviewed by experts, an IF diet was presented to the children participating in the research. Parents expressed interest in the diet and even suggested that they be given the diet to use at home. A similar approach was used in this study wherein expert consultation was included during all stages of model development in the form of meetings and workshops. In addition, results that were suitable to fit into this model derived from stakeholder discussions that took place during data collection was added. Children and the community accepted the intervention meals made from local vegetables and parents requested to be shown how to prepare these meals at home. This supports the belief that expert consultation and stakeholder engagement are crucial for behavior change or when you introduce something new (41).

This model emphasizes that collaboration with chefs is crucial when promoting locally available foods among children or the public. In this study, the chef innovated with the ingredients from *Amaranthus*, *Bidens Pilosa*, and *Vigna Unguiculata*, developed appealing and tasty recipes for children, and used the school meals without changing them; dietary diversification was therefore promoted. This is supported by Borelli et al. (31) who prepared meals in collaboration with local communities and produced a recipe book. The economic benefit was activated through supporting local small-scale farmers who provided the vegetables throughout the implementation of the intervention. Furthermore, Naidoo and Wills (30) assert that making healthy choices easier denotes how the environment, social networks, sense of security, socio-economic circumstances, families, and resources individuals have in their local neighborhood can affect their health. In this study peers, parents, schoolteachers, and representatives from the Vhembe NSNP team were receptive to the initiation and accepted the intervention meal; this could easily promote and enhance societal change. The one limitation is the validation of the model by end-users, done virtually by experts during a workshop at first, then the second they were given a tool and the model to validate. There was, however, a poor response rate from the identified end-users. Another limitation is that the intervention was conducted in a rural based school, we do not know if the same success would have been observed in urban areas. The strengths include the use of participatory approaches to engage end-users and experts to develop the model.

## Conclusion and recommendations

This study’s primary aim was to develop and validate a model for the consumption of locally available vegetables using the intervention dishes (*Amaranthus* cabbage, *Bidens* dumpling, and Fishpea), specifically for primary school children in the Vhembe District of the Limpopo Province. The model developed and implemented through this research suggests that it is possible to promote the consumption of locally available vegetables among primary school children if innovative ways to develop attractive recipes are used. These recipes are likely to be accepted and consumed by children and then must be expanded to include primary school children throughout the community and into the rest of the country. This research demonstrates that children can be and must be engaged as effective change agents, especially where their health and the health of their communities are concerned. The study recommends that many key role players must be involved in primary school children’s health and nutrition intake – it is an effort that requires many different areas of expertise, experience, and action. The developed model was validated by experts and end-users and found to be implementable, understandable, and relevant to the context.

## Data availability statement

The original contributions presented in the study are included in the article/supplementary material, further inquiries can be directed to the corresponding author/s.

## Ethics statement

The studies involving humans were approved by Health Research Ethics Committee of Stellenbosch University. The studies were conducted in accordance with the local legislation and institutional requirements. Written informed consent for participation in this study was provided by the participants’ legal guardians/next of kin.

## Author contributions

HM: Conceptualization, Formal analysis, Funding acquisition, Investigation, Methodology, Writing – original draft. BT: Conceptualization, Formal analysis, Investigation, Writing – review & editing. MZ: Methodology, Supervision, Writing – review & editing. XM: Funding acquisition, Supervision, Visualization, Writing – original draft, Writing – review & editing.

## Footnotes

^1^*Spaza* refers to a convenience shop in a residential area.
